# Enrichment of a neutrophil-like monocyte transcriptional state in glioblastoma myeloid suppressor cells

**DOI:** 10.21203/rs.3.rs-3793353/v1

**Published:** 2023-12-28

**Authors:** J.K. Wiencke, Emily Nissen, Devin C. Koestler, Stan J. Tamaki, Courtney M. Tamaki, Helen M. Hansen, Gayathri Warrier, Sara Hadad, Lucie McCoy, Terri Rice, Jennifer Clarke, Jennie W Taylor, Lucas A. Salas, Brock C. Christensen, Karl T. Kelsey, Rondi Butler, Annette M. Molinaro

**Affiliations:** 1 Department of Neurological Surgery, University of California San Francisco, San Francisco, CA.; 2 Department of Neurology, University of California San Francisco, San Francisco, CA; 3 Parnassus Flow Cytometry CoLab, University of California San Francisco, San Francisco, CA 94143-0511, USA; 4 Department of Epidemiology and Biostatistics, University of California San Francisco, San Francisco, CA.; 5 Department of Epidemiology, Geisel School of Medicine, Dartmouth College, Lebanon, NH.; 6 Department of Biostatistics & Data Science, University of Kansas Medical Center, Kansas City, KS.; 7 Department of Molecular and Systems Biology, Geisel School of Medicine, Dartmouth College, Lebanon, NH.; 8 Department of Community and Family Medicine, Geisel School of Medicine, Dartmouth College, Lebanon, NH.; 9 Department of Epidemiology, Brown University, Providence, RI.; 10 Department of Pathology and Laboratory Medicine, Brown University, Providence, RI.

## Abstract

Glioblastomas (GBM) are lethal central nervous system cancers associated with tumor and systemic immunosuppression. Heterogeneous monocyte myeloid-derived suppressor cells (M-MDSC) are implicated in the altered immune response in GBM, but M-MDSC ontogeny and definitive phenotypic markers are unknown. Using single-cell transcriptomics, we revealed heterogeneity in blood M-MDSC from GBM subjects and an enrichment in a transcriptional state reminiscent of neutrophil-like monocytes (NeuMo), a newly described pathway of monopoiesis in mice. Human NeuMo gene expression and Neu-like deconvolution fraction algorithms were created to quantitate the enrichment of this transcriptional state in GBM subjects. NeuMo populations were also observed in M-MDSCs from lung and head and neck cancer subjects. Dexamethasone (DEX) and prednisone exposures increased the usage of Neu-like states, which were inversely associated with tumor purity and survival in isocitrate dehydrogenase wildtype (IDH WT) gliomas. Anti-inflammatory *ZC3HA12/Regnase-1* transcripts were highly correlated with NeuMo expression in tumors and in blood M-MDSC from GBM, lung, and head and neck cancer subjects. Additional novel transcripts of immune-modulating proteins were identified. Collectively, these findings provide a framework for understanding the heterogeneity of M-MDSCs in GBM as cells with different clonal histories and may reshape approaches to study and therapeutically target these cells.

## INTRODUCTION

The concept of an immunosuppressive network operating in the tumor microenvironment that impacts hematopoiesis and circulating immune compartments is well established across many cancer types^1^. The GBM immune landscape is dominated by myeloid-derived cells^2^. While the cancer is confined to the central nervous system, GBM patients display systemic immune defects^3^. Central players in this network are bone marrow-derived polymorphonuclear and mononuclear myeloid cell populations^4–6^. Among these are monocytes (Lineage^negative^/CD33^+^/CD14^+^ cells) that lack cell surface expression of major histocompatibility complex (MHC) class II proteins (e.g., HLA-DR) and that inhibit T cell response *in vitro*. These cells have been widely studied and termed monocytic myeloid-derived suppressor cells (M-MDSC)^7^. There is strong support for the association of flow-cytometrically (FCM) defined M-MDSCs with poor survival and tumor resistance to radiation^8^ and immunotherapies^9,10^. Lack of HLA class II expression reflects monocyte dysfunction^11^, reduced responsiveness to microbial stimuli^12^, and is associated with T cell immunosuppression^13^. In human GBM, expansion of putative immunosuppressive myeloid cells, including M-MDSCs, has been documented^14–19^, and their numbers were increased in the blood of subjects exposed to the synthetic glucocorticosteroid, dexamethasone (DEX)^14,20^. High levels of M-MDSCs in recurrent GBM tumor tissue have been associated with poor survival^17^. The abundance of monocytic MDSCs has also been reported to be prognostic in infectious diseases, including bacterial sepsis^21–23^ and, most recently, COVID-19^24,25^. In the latter, FCM-measured M-MDSC frequencies, early in the course of infection, were strongly associated with disease severity and T cell suppression, indicating an essential role for M-MDSCs in the dysregulated COVID-19 immune response^26^. Other researchers confirmed that elevated burdens of HLA-DR negative monocytes were strongly related to immunosuppression and poor COVID-19 survival^27^. The involvement of M-MDSCs in diverse pathological conditions, including cancer and infectious diseases, underscores the broad impact of myeloid cell populations as moderators of immune response and outcome. These findings also highlight the substantial therapeutic opportunities that could be created by monitoring and modulating MDSC biology^28–31^.

Despite extensive clinical support for M-MDSC as essential markers of pathology, there are still significant gaps in our understanding of the origins and phenotypic characteristics of these cells^32^. The ontogeny of M-MDSCs is unknown, and in most studies, definitive evidence of T cell suppression by *in vitro* assays is not assessed. Even when observed, the potential contributions of cell heterogeneity in bulk proliferation assays cannot be ascertained^33^. Thus, the M-MDSC designation has been viewed as ambiguous and self-limiting^13,34^. Beyond absence of HLA-DR expression, no consensus exists on specific M-MDSCs markers, although the ectoenzyme Vannin-2/*VNN2*^35,36^, alarmin proteins *S100A8*, *S100A9*, and *S100A12*^37^, *CXCR1*^38^, and annexin-A1 (*ANXA1*)^39^ have been proposed. The lack of specific M-MDSC markers is a barrier to both improved prognostication and the development of therapeutics to mitigate myeloid immunosuppression. In a search for “internal” markers of MDSCs, which do not rely on cell surface expression, investigators have examined altered DNA methylation^40^ and transcriptional signatures^26,41,42^.

Recent single-cell transcriptomic studies (scRNA-seq) have shed light on the complex landscape of the myeloid cell space and challenge the conventional linear model of monopoiesis. This model traditionally follows a progression from common myeloid progenitors (CMP) to classical monocytes through granulocyte-macrophage progenitors (GMPs), monocyte dendritic cell progenitors (MDPs), and ultimately a restricted common monocyte progenitor (cMoP)^43–45^. In contrast, combining scRNA-seq and lineage tracing in mice revealed two routes of monocyte differentiation that leave an imprint on mature cells^43,46,47^. The first ontogenetic pathway led to a neutrophil-like monocyte (Neu-like) that was proposed to arise from GMP cells, whereas the second derived from MDP cell progenitors and gave rise to a dendritic cell-like monocyte (DC-like). Other researchers have questioned the relevance of the MDP population in the production of neutrophil-like monocytes^48^. Gene markers of alternate developmental pathways of human monocytes have been suggested^47^, and multiple scRNA-seq studies support the existence of distinct transcriptional states that resemble previously described neutrophil-like and dendritic-like murine monocytes. In a seminal study of healthy blood donors, Villani et al.^49^ found a novel monocyte population, cluster “Mono3”, that was distinguished from classical and non-classical subtypes. In COVID-19 subjects, and consistent with the earlier single-cell analysis^49^, Silvin et al.^50^ reported the presence of a novel monocyte cluster (cluster “hMono3”) that expressed a set of neutrophil-associated genes, including *S100A8*/*S100A9* and colony-stimulating factor 3 receptor (*CSF3R*); the latter being an essential growth factor receptor for polymorphonuclear phagocytes. Mulder et al.^51^ assembled a comprehensive atlas of tissue and circulating mononuclear phagocytes that revealed six monocyte populations, including one with low or negative HLA-DRB1 mRNA expression (cluster #8; *CD14*^*+*^*/S100A8/S100A9/S100A12*^*hi*^) that was mapped to subjects with severe COVID-19 in a reanalysis of the Silvin et al. data^50^. Schulte-Schrepping et al.^52^ identified six monocyte populations and found one with a gene signature reminiscent of the earlier reported classical monocyte expressing neutrophilic genes (cluster 0; HLA-DR^low^, *S100A8/S100A9/S100A12*^*high*^). Cluster 0 cells accumulated during viral infection and were sustained in subjects suffering severe but not mild COVID-19 disease^52^. Thus, the innate immune system, mainly monocytes, is linked to the heterogeneity of the COVID-19 disease course. In systemic bacterial infection, Reyes et al.^53^ identified a blood CD14^+^ monocyte state they termed MS1, which was closely associated with sepsis in multiple cohorts. The MS1-B subcluster exhibited high *S100A8/S100A12* and *VNN2* expression, previously implicated as an M-MDSC markers in glioma^36^. In lung cancer tissue and blood, Zilionis^54^ described a subtype of classical monocytes (termed hbMono3: blood; hMono3: tissue) that uniquely expressed a set of neutrophil-associated genes, including *S100A8/S100A9* and *CSF3R.* The hbMono3 transcriptional signature was associated with shorter survival times and was conserved in mouse blood and human lung tumor infiltrates. Finally, scRNA-seq analysis of GBM tumor tissues revealed five myeloid cell signatures and three (MC2–MC5, and MC7) as independent prognostic indicators of patient survival^55^ The ontogenic relationships among these novel monocyte-related transcriptional states across different studies or to FCM-gated M-MDSCs are poorly defined.

The association of several M-MDSC features with putative Neu-like monocyte phenotypes led us to compare M-MDSC gene expression in GBM subjects with an assemblage of the aforementioned single-cell mononuclear signatures and with our scRNA-seq data from isolated M-MDSC and monocytes from GBM subjects. Our results indicate the enrichment of a novel transcriptional state resembling an alternate pathway of monocyte development. Subclusters within this state were marked by potentially drug-able immunoregulatory targets, thus providing a new framework to discern the heterogeneity of M-MDSCs.

## RESULTS

### M-MDSCs from GBM subjects display differentially expressed genes.

**Figure 1.**
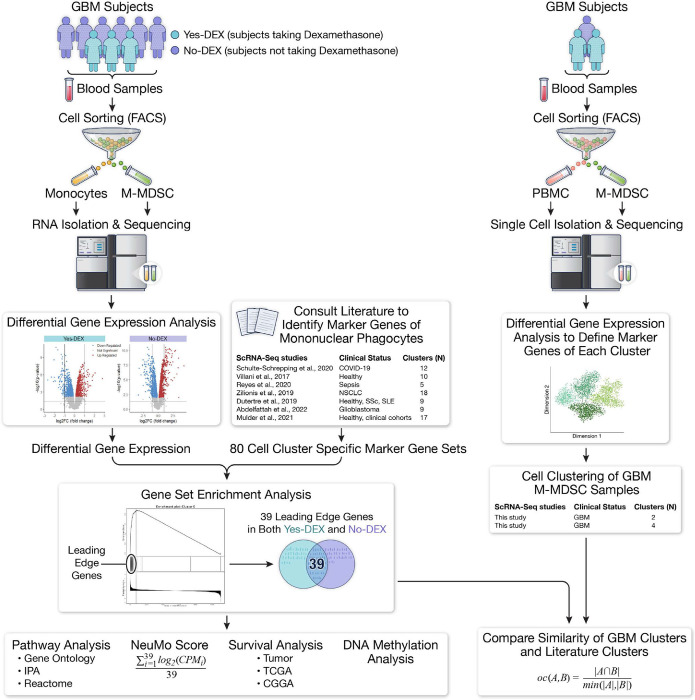
Graphical summary of study design to identify M-MDSC differentially expressed genes and their associations with novel myeloid transcriptional states and clinical outcomes.

**Figure 2:**
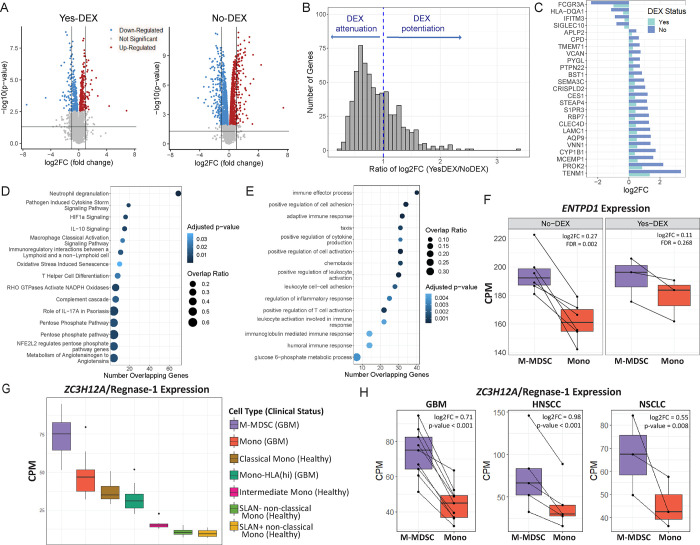
Bulk RNA-seq analyses of M-MDSC and paired HLA-DR^+^ monocytes (Mono) from GBM, head and neck and lung cancer subjects. **A**. Volcano plots visualizing the results of the differential expression analysis for Yes-DEX (N=3) and No-DEX (N=6) paired M-MDSC and Mono. The horizontal black line represents a p-value of 0.05. Each point represents a gene. Red indicates the up-regulation of the gene in M-MDSC compared to monocytes (log_2_FC>0, FDR<0.05) and blue indicates down-regulation (log_2_FC<0, FDR<0.05). **B**. Histogram showing the distribution of the ratio of log_2_FC in the 666 DEGs in common and regulated in the same direction between Yes-DEX and No-DEX. **C**. Bar plot of the 25 DEGs with the most considerable DEX mediated attenuation. The x-axis is the log_2_FC from the differential expression test. A teal bar indicates the Yes-DEX group, and a light blue bar indicates the No-DEX group. **D and E**. Dot plot showing top 15 significant Ingenuity Pathways (D) and GO Biological Processes (E) from an over-representation analysis of DEX attenuated genes. The x-axis is the number of DEX attenuated genes that overlap with the pathway or GO term. The size of the dot reflects the magnitude of the overlap (i.e., Number of Overlapping Genes/Total Number of Genes in Pathway), while the color represents significance from the over-representation test. **F**. Boxplots of *ENTPD1* in paired M-MDSC (purple) and Mono (red) in No-DEX and Yes-DEX groups. The y-axis is counts per million (CPM). Black lines connect M-MDSC and Mono from the same individual. The log_2_FC and FDR values are from the differential expression test in A. **G**. Boxplots of *ZC3H12A/Regnase-1 expression* in M-MDSC (purple, N=9), Mono (red, N=10), Mono-HLA(hi) (dark green, N=8) from GBM patients and Classical Mono (brown, N=8), Intermediate Mono (pink, N=8), SLAN− non-classical Mono (light green, N=8) and SLAN+ non-classical Mono (yellow, N=8) from healthy individuals. The y-axis is in CPM. **H**. Boxplots of *ZC3H12A/Regnase-1* in paired M-MDSC (purple) and Mono (red) across 3 studies: GBM (this study), HNSCC (GSE183854), and NSCLC (GSE162353). Black lines connect paired samples. The y-axis is in CPM.

Demographic and DEX exposure data are shown in Supplementary Table 1. Differentially expressed genes (DEGs) between paired peripheral blood M-MDSC and monocyte samples using bulk RNA-seq were determined separately for GBM patients who were dexamethasone (DEX) exposed and non-exposed (Fig. 1) at the time of blood draw. Some subjects classified as non-exposed had previous exposures (Supplementary Table 1). M-MDSC expressed low but detectable levels of HLA-DR and other MHC transcripts compared to paired monocytes (Supplementary Fig. 1). The Yes-DEX samples had 422 up-regulated and 356 down-regulated genes in M-MDSCs compared to monocytes (Fig. 2a, Supplementary Table 2). The No-DEX samples had 1637 up-regulated and 1478 down-regulated genes (Fig. 2a, Supplementary Table 2). There were 667 DEGs in common between Yes-DEX and No-DEX comparisons, with the No-DEX having 2448 unique DEGS and Yes-DEX having 111 unique DEGs. There were 40 overrepresented Ingenuity pathways in common to both Yes-DEX and No-DEX (Supplementary Fig. 2).

### Dexamethasone predominately attenuates differential gene expression in M-MDSCs

We found that differential expression in DEGs was predominately attenuated by comparing fold changes in gene expression in DEX-exposed to non-exposed subjects. That is, of the 666 DEGs with log_2_ fold changes (FC) in the same direction between the Yes-DEX and No-DEX groups a majority (67%) of the Yes-DEX log_2_-FC values for a gene were markedly lower than the No-DEX log_2_FC, which we term DEX attenuation. To characterize the DEX attenuation effect in DEGs, we defined the ratio of log_2_FCs. This statistic is simply the Yes-DEX log_2_-FC for a gene divided by the No-DEX log_2_FC for the same gene; thus, the statistic is a positive number, and a ratio between 0 and 1 indicates DEX attenuation of expression of a gene. There were 447 DEGs that exhibited DEX attenuation (Fig. 2b). The 25 DEGs with the most considerable DEX attenuation (i.e., the smallest ratio of log_2_FCs) are shown in Fig. 2c. To assess the chance of observing 447 genes with DEX attenuation, we derived the distribution of genes with DEX attenuation under the null hypothesis. Our observed value of 447 genes is far above the range of this distribution [216, 323], thus indicating that the number of genes with DEX attenuation is higher than expected. We performed an overrepresentation analysis using the DEX attenuated genes. We found they are enriched in Ingenuity pathways (Fig 2d) and Gene Ontology (GO) biological processes (Fig 2e) such as neutrophil degranulation and immune effector process. An instructive example of attenuation is seen in *ENTPD1,* in which the DEX attenuation leads to a loss in differential expression. That is, a greater abundance of *ENTPD1* transcripts in M-MDSC compared to paired monocytes in No-DEX subjects (log_2_FC = 0.27, FDR = 0.002) were observed, but not in Yes-DEX subjects (log_2_FC = 0.11, FDR = 0.268), giving a ratio of log_2_FCs of 0.41. (Fig. 2f).

### Differential expression of candidate immune modulatory transcripts in GBM M-MDSCs

The abundance of 56 gene transcripts in recognized immunomodulatory pathways was evaluated (Supplementary Table 3) in the M-MDSCs and monocytes. Lower expression (compared with monocytes) of several costimulatory transcripts (*CD86, LGALS9, ICOSLG, B7-H6*) was observed. No evidence of significant overexpression was found (FDR>0.05) for many of the classic immune checkpoint genes (e.g., *PD1, PDL1, CTLA4, LAG3, TIGIT*) or proposed MDSC immunosuppressive effector genes (e.g., *ARG1, IDO1, NOS2*). Many had non-detectable transcript levels. Overexpressed genes in No-DEX samples included *ZC3H12A*/*Regnase-1*, *TNFAIP3, ENTPD1, SIRPA, ADAM17* and *RC3H1*, whereas in Yes-DEX only *ZC3H12A*/*Regnase-1* and *TNFAIP3* were significantly overexpressed. Regnase-1 expression was examined across M-MDSCs and monocytes (Fig. 2g). M-MDSCs showed the highest levels of expression, followed by monocytes from GBM patients and classical monocytes from healthy individuals. Intermediate monocytes and non-classical monocytes show the lowest levels of Regnase-1 expression. We also studied Regnase-1 expression in three datasets with paired M-MDSC and HLA-DR^+^ monocyte samples: GBM (this study), head and neck squamous cell cancer (HNSCC), and non-small cell lung cancer (NSCLC). Across all three, M-MDSC consistently showed higher expression of Regnase-1 (Fig. 2h). To compare the effect of change in expression, a metric called the equivalent change index (ECI) was used. The ECI of Regnase-1 for the pairwise comparisons of the three studies were greater than 0.5. Change in expression of Regnase-1 between M-MDSC and monocytes was most equivalent between GBM and HNSCC (ECI=0.77) and GBM and NSCLC (ECI=0.73).

### Gene set enrichment analysis of scRNA-seq myeloid cell populations

**Figure 3:**
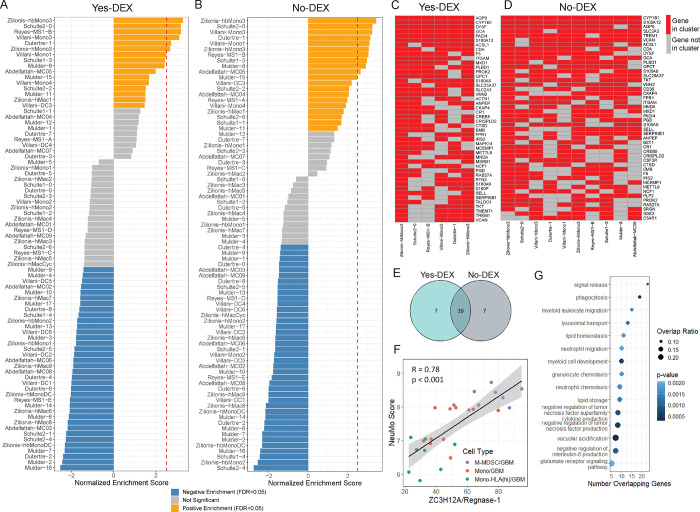
Identification of genes enriched in M-MDSCs and creating a NeuMo expression score that includes overlapping genes in DEX exposed and non-exposed subjects. **A and B**. Gene Set Enrichment Analysis (GSEA) results for Yes-DEX (A) and No-DEX (B) samples. The y-axis is the name of the scRNA-seq cluster derived from the literature. The x-axis is the normalized enrichment score (NES). The bar is colored in orange for “Positive Enrichment” (FDR<0.05, NES>0). This indicates a scRNA-seq cluster is overrepresented at the genes up-regulated in M-MDSC compared to Mono. The bar is blue for “Negative Enrichment” (FDR<0.05, NES<0). This indicates a scRNA-seq cluster is overrepresented at the down-regulated genes in M-MDSC compared to Mono (i.e., up-regulated in Mono). The bar is colored in grey if FDR>0.05. The red dashed line is at a NES=2.5. **C and D**. Heatmaps of the most common leading-edge genes among the 6 scRNA-seq literature-derived clusters from the Yes-DEX GSEA (C) and the 10 scRNA-seq literature-derived clusters from the No-DEX GSEA (D), genes on the y-axes and scRNA-seq clusters on the x-axes. These 6 and 10 gene sets were chosen due to their high NES (>2.5) and low FDR (<0.05). Red boxes denote genes found to be in the leading-edge for that cluster from the GSEA, and grey if not. **E**. A Venn diagram of the overlap between Yes-DEX, No-DEX leading-edge genes. **F**. Scatter plot of NeuMo score versus *ZC3H12A* expression in M-MDSC, Mono, and Mono-HLA (hi) from GBM samples, where “R” is the Pearson correlation coefficient, and the black line is the best fit line. **G**. Dot plot showing the results of a pathway enrichment analysis using Gene Ontology (GO) Biological Processes terms for the NeuMo (39) and NeuMo-correlated genes (531). The y-axis contains the name of the GO term and the x-axis, the number of input genes NeuMo and NeuMo-correlated genes that overlap with the GO term. The size of the dot reflects the magnitude of the overlap (i.e., Number of Overlapping Genes/Total Number of Genes in Pathway), while the color represents significance from the over-representation test.

Seven single-cell expression reports were reviewed^49–54^, from which 80 signature gene sets of monocytic phagocyte populations were collated (Supplementary Table 4). The studies encompassed cells from healthy donors, COVID-19, bacterial sepsis, and lung cancer subjects. We included one study of resident and bone marrow-derived cells isolated from GBM tumor tissue^55^. Given their association with neutrophil-like monocytes, we noted 15 of the signature gene sets included *S100A8, S100A9, S100A12*, or *VNN2* and the putative neutrophil-like monocyte phenotype (NeuMo). To examine the similarity of M-MDSC genes identified from our bulk RNA-seq and these signature gene sets, we conducted a gene set enrichment analysis (GSEA). The GSEA identifies which of the 80 signature gene sets are enriched with up-regulated genes (i.e., positive enrichment, normalized enrichment score (NES)>0) or down-regulated genes (i.e., negative enrichment, NES<0) in M-MDSC compared to monocytes from our bulk RNA-seq data. We chose the signature gene sets with an NES>2.5 to determine which contain genes with the most highly up-regulated in M-MDSCs compared to monocytes in bulk RNA-seq. Six gene sets and ten gene sets for the Yes-DEX and No-DEX groups, respectively, passed this threshold (Figs. 3a, 3b).

### Creating the neutrophil-like monocyte (NeuMo) expression scores

The leading-edge genes from the GSEA for the six positively enriched gene sets in Yes-DEX (Supplementary Table 5) and ten positively enriched gene sets in No-DEX (Supplementary Table 6) were compared to find similar genes that were also defining genes of M-MDSCs across all the signature gene sets. There were 39 leading-edge genes present in a majority of the selected positively enriched gene sets in both Yes-DEX and No-DEX (Figs. 3c, 3d). The 39 in common genes were used to create a NeuMo metagene expression score (Fig. 3e, Supplementary Table 7). The NeuMo score represents the average expression in a sample across the 39 genes. We also identified an expanded NeuMo gene set by selecting genes with high correlation (R>0.7) with the 39 gene NeuMo score in monocytes and matched M-MDSCs to take advantage of the deeper sequence depth of bulk RNA sequencing. An additional 531 genes met these criteria (Supplementary Table 8), including Regnase-1 (R=0.78, Fig 3f). This expanded NeuMo gene set is enriched in GO biological processes such as signal release, phagocytosis, and myeloid leukocyte migration (Fig 3g).

### Increased NeuMo expression scores in cancer subjects

**Figure 4:**
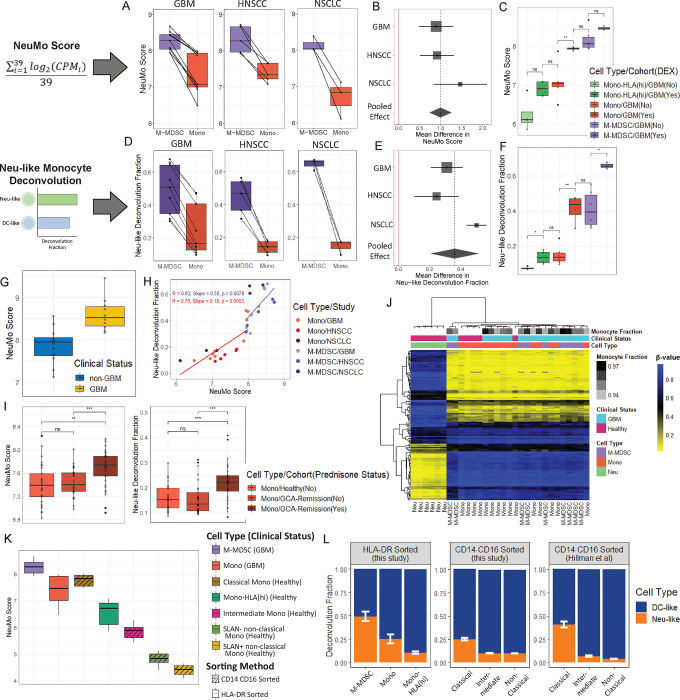
Assessment of the NeuMo score and Neu-like monocyte deconvolution fraction in bulk RNA-seq cancer and glucocorticoid exposure datasets. **A and D.** NeuMo score (A) and Neu-like deconvolution fraction (D) in paired M-MDSC (purple) and monocytes (red) from GBM, HNSCC and NSCLC. **B and E**. Meta-analysis of NeuMo score (B) and Neu-like fraction (E). The points represent the mean difference between M-MDSC and monocytes, with the stippled lines (or diamond width) representing the 95% CI and the box size representing the sample size. **C and F**. NeuMo scores (C) and Neu-like fractions (F) split by DEX status in monocytes, HLA-DR high monocytes and M-MDSC. **G**. NeuMo score in whole blood from GBM (N=10) and non-GBM (N=12) individuals. **H**. Scatter plot of NeuMo score and Neu-like fraction. “R” depicts Pearson’s correlation coefficient. **I**. NeuMo score and Neu-like fractions in monocytes from prednisone treated giant cell arteritis (GCA) subjects, untreated GCA subjects, and controls^57^. **J**. Heatmap of monocyte and neutrophil lineage-discriminating CpG probes for M-MDSC from GBM (N=9), Mono from GBM (N=10) and healthy donors (N=5) and neutrophils (Neu) from healthy individuals (N=6). The colors within the heatmap represent the beta-value ranging from 0 (yellow) to 1 (blue). The monocyte fraction is estimated from CIBERSORTx using expression data. A monocyte fraction was not estimated for healthy Mono or Neu (colored in white). **K**. NeuMo score in M-MDSC (N=9), Mono (N=10), Mono-HLA (hi) (N=8) from GBM patients and Classical Mono (N=8), Intermediate Mono (N=8), SLAN− non-classical Mono (N=8) and SLAN+ non-classical Mono (N=8) from healthy individuals. Striped boxplots indicate sorting based on CD14 CD16 and no stripes indicate sorting based on HLA-DR. **L**. Average deconvolution fraction of Neu-like (orange) and DC-like (blue) cell states on bulk RNA-sequenced M-MDSC, Mono, Mono-HLA(hi) from GBM patients (this study), classical, intermediate, non-classical monocytes (SLAN− and SLAN+ were grouped together) from healthy individuals (this study), and classical, intermediate, and non-classical monocytes in another healthy individual cohort^59^. Error bars represent the standard error of the mean of the Neu-like fraction. P-values in boxplots in C and F are based on Wilcoxon rank-sum test and in I on two-sample t-test: ns=p>0.05, *=p<0.05, **=p<0.01, ***=p<0.001

The NeuMo score was compared in paired M-MDSC and monocyte samples from three cohorts: GBM (this study), HNSCC (GSE183854), and NSCLC (GSE162353). The NeuMo score was significantly higher in M-MDSCs compared to monocytes from individuals in all three cohorts (Fig. 4a, 4b). Performing a meta-analysis, with a random-effects model, the pooled effect size was a mean difference in M-MDSC and monocytes of 1.00 (95% CI = [0.74, 1.26]) (Fig. 4b). In a study of GBM patients, the NeuMo score was significantly higher in whole blood from GBM patients compared to non-GBM donors (Δ =0.74, p = 0.0002, Fig. 4g), even after adjustment for neutrophil levels (Δ = 0.55, p = 0.005).

### Enriched fractions of Neu-like monocytes in M-MDSC

To provide a complementary and independent approach to evaluate neutrophil transcriptional state in M-MDSCs and monocytes, samples were deconvoluted with a semi-supervised non-negative matrix factorization (NMF) algorithm using a guide matrix of published marker genes for Neu-like monocytes and DC-like monocytes^47^ (Figs. 4d, 4e, 4f). The Neu-like fraction was significantly higher in M-MDSCs than in monocytes from individuals with GBM, HNSCC, and NSCLC (Figs. 4d, 4e). Again, a meta-analysis was performed, with a random-effects model that revealed a pooled effect size difference of 0.36 (95% CI = [0.21, 0.52]) (Fig. 4e). The NeuMo score and Neu-like fraction were positively correlated in both M-MDSC (R=0.62) and Mono (R=0.72), however their slopes were not significantly different (p = 0.22) (Fig. 4h).

### Glucocorticoid exposure expands the neutrophil-like transcriptional state.

The NeuMo score and Neu-like fraction were consistently increased in M-MDSC and monocytes from Yes-DEX samples (Figs 4c, 4f). Although for the NeuMo score, one sample in the No-DEX group was an outlier. This sample came from an individual who was DEX exposed 15 days prior. Thus, we also compared samples from individuals DEX exposed at blood draw (i.e., the original Yes-DEX) and those who have not been exposed to DEX in the prior month (N=4, Supplementary Table 1). The NeuMo score in this comparison was significantly higher in Yes-DEX samples (Δ = 0.46, p = 0.0002). In a study of giant cell arteritis (GCA)^57^, the NeuMo scores were increased in monocytes from GCA subjects exposed to prednisone compared to monocytes from healthy individuals (Δ = 0.288, p = 0.001) and to monocytes from individuals with GCA not exposed to prednisone (Δ = 0.243, p = 0.0005) (Fig. 4i). The Neu-like fraction was also elevated in prednisone exposed subjects (Fig. 4i).

### M-MDSCs display canonical transcriptional and epigenetic features of monocytes.

Given that M-MDSCs are enriched with neutrophil-related transcripts, we asked whether they also display canonical neutrophilic epigenetic or gene expression features. Using the lineage discriminating CpG probes that drive methylation deconvolution^58^, we observed that M-MDSCs clustered tightly with monocytes rather than neutrophils (Fig. 4j). CIBERSORTx deconvolution of gene expression was concordant with the DNA methylation results, indicating monocyte identity of isolated M-MDSCs in GBM subjects irrespective of DEX status. CIBERSORTx predicted all samples to have a monocyte fraction greater than 93%, and all samples had a 0% predicted neutrophil fraction (Supplementary Table 9).

### Significant differences of Neu-like expression in classical, intermediate, and non-classical monocytes.

Because M-MDSCs are isolated and identified through differential expression of MHC class II surface expression and conventional monocyte subtypes by their differential expression of CD14 and CD16, we estimated NeuMo scores and Neu-like fractions of classical, intermediate, and non-classical monocytes from healthy individuals (Figs 4k, 4l). M-MDSCs had the highest NeuMo scores and Neu-like fractions compared to all other monocyte subtypes. Non-classical cells exhibited the lowest NeuMo scores and Neu-like fractions (p<.0001, Supplementary Table 17). We confirmed the markedly lower NeuMo scores and Neu-like deconvolution fraction in non-classical compared to classical monocytes in an independent data set of healthy control blood donors (Supplementary Table 17)^59^.

### NeuMo scores and Regnase-1 expression are elevated in IDH WT compared with IDH MT tumors and associated with low tumor purity and poor survival.

**Figure 5:**
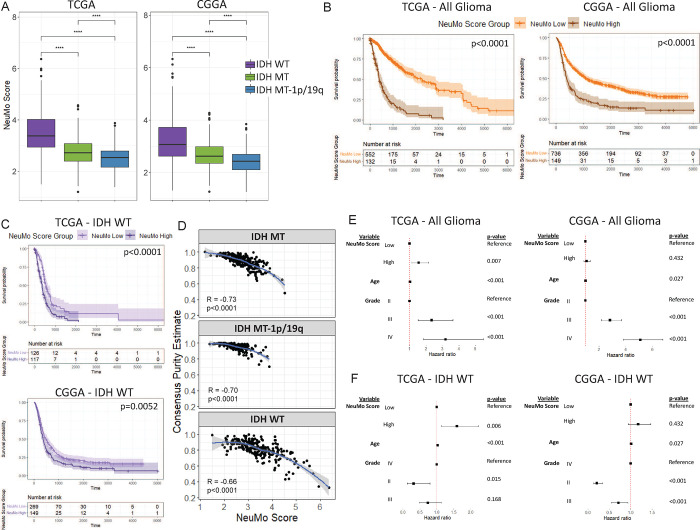
Association of tumor NeuMo score with glioma molecular subtype, tumor purity and survival in TCGA and CGGA. **A.** Boxplot of NeuMo score across tumor samples of isocitrate dehydrogenase wild type (IDH WT) (N=243 and N=418), IDH mutant (IDH MT) (N=270 and N=296), IDH MT-1p/19q codeletion (i.e. oligodendroglioma) (N=171 and N=171) from the TCGA and CGGA. P-values based on a two-sample t-test: ns=p>0.05, *=p<0.05, **=p<0.01, ***=p<0.001, ****=p<0.0001 **B and C**. Kaplan Meier plots showing survival probability of all glioma samples (B) and only IDH WT samples (C) in the TCGA and CGGA datasets. Groups are split into those with high (brown) or low (orange) NeuMo score for all glioma (B). Groups are split into high (dark purple) or low (light purple) NeuMo score for only IDH WT (C). P-value is based on log-rank test. **D**. Scatter plot showing the inverse correlation between NeuMo score and consensus purity estimate (CPE) in the TCGA, stratified by IDH mutation status. Loess regression line shown with “r” (Spearman correlation coefficient) and associated p-value. **E and F**. Forest plots showing results from multivariable Cox PH models in the TCGA and CGGA – all glioma (E) (these models are also adjusted for IDH group, added as a strata in model) and for IDH WT only (F).

Using TCGA and CGGA data, we estimated NeuMo scores in glioma samples. We observed higher scores among IDH WT tumors compared to IDH mutant tumors (Fig. 5a). The NeuMo score was dichotomized into a high NeuMo score group and a low NeuMo score group, using a cutpoint/threshold determined in the TCGA data only. Among all grades and mutation groups of glioma, a high NeuMo score was associated with shorter survival in the TCGA (HR = 5.18, 95% CI = [3.99, 6.72], Fig. 5b). The CGGA was used as a validation set, and among all grades and mutation groups of glioma, we also saw a high NeuMo score was associated with shorter survival (HR = 2.14, 95% CI = [1.75, 2.62], Fig. 5b). In a similar analysis, using only IDH WT tumors, a high NeuMo score was associated with worse survival in both the TCGA (HR = 1.93, 95% CI = [1.43, 2.62]) and the CGGA (HR = 1.37, 95% CI = [1.10, 1.70]) datasets (Fig. 5c). The TCGA also had estimates of tumor purity, which was inversely associated with NeuMo score (Fig. 5d). In multivariate analysis of all glioma samples a high NeuMo score remained significantly associated with shorter survival, when adjusted for IDH status (as a strata), grade, and age in TCGA (HR = 1.56, 95% CI = [1.13, 2.15]). In the same model in the CGGA, a high NeuMo score remained associated with shorter survival, but was not significant (HR = 1.09, p=0.43) (Fig. 5e). In the TCGA, we also fit a model adjusting for tumor purity, however, both NeuMo score (HR = 1.413, 95% CI = [0.96, 2.07]) and tumor purity (HR = 0.41, 95% CI = [0.09, 1.82]) became not significant. This was similar in a multivariate analysis of only IDH WT tumor samples (Fig 5f). *TNFAIP3* and *Regnase-1* expression was significantly higher in TCGA and CGGA IDH WT tumors compared to IDH mutant tumors (Supplementary Figs. 3a, 3b), and the expression of each of these two genes was correlated with the NeuMo score (Supplementary Figs. 3c, 3d). *Regnase-1* was also inversely correlated with tumor purity (Supplementary Fig. 3e). In IDH WT tumors, higher *Regnase-1* expression was associated with worse survival (Supplementary Fig. 3f).

### scRNA-seq reveals novel NeuMo and DC transcriptional states in M-MDSC from GBM subjects.

**Figure 6:**
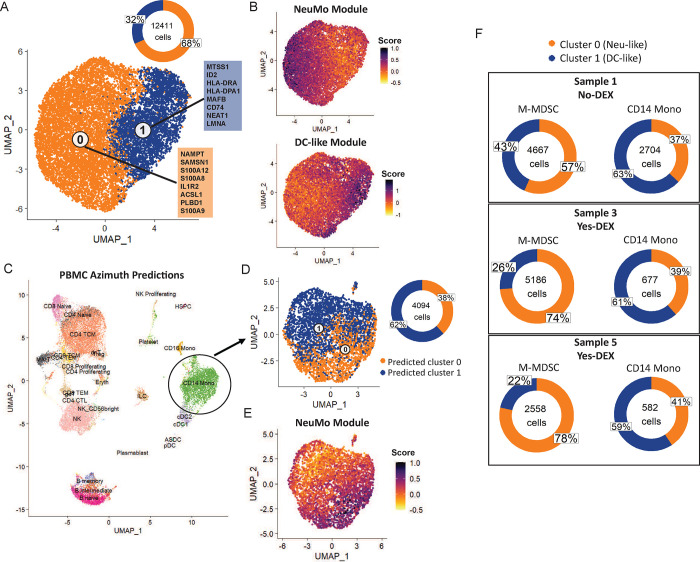
GBM scRNA-seq clusters at 2-compartment resolution and their prevalence in M-MDSC and classical, intermediate and non-classical monocytes. **A.** Integrated and clustered M-MDSC samples (N=3). The orange (cluster 0, Neu-like state) and blue (cluster 1, DC-like state) boxes represent each cluster’s top 8 marker genes by log_2_FC. Donut plot indicates the proportion of cells in each of the two clusters. **B**. NeuMo and DC-like module scores for the M-MDSC integrated data. A darker purple indicates a higher score (i.e., increased expression of NeuMo-associated or DC-associated genes) and a yellow color indicates a lower score. **C**. Integrated and clustered PBMC samples from healthy donors (N=4) with cells colored in by Azimuth cell type predictions. **D.** Integrated and clustered predicted CD14+ monocytes from PBMC. A cell’s cluster classification was predicted using the M-MDSC clusters in (A) as the reference. **E.** NeuMo module score for the CD14+ monocytes. A darker purple color indicates a higher score. **F.** Donut plots comparing proportion of Neu-like and DC-like cells between M-MDSC and CD14+ monocyte samples from the same individual. The proportions are calculated by splitting the integrated M-MDSC data (A) and integrated CD14+ monocyte data (D) by individual.

We applied 10x scRNA-seq on isolated M-MDSC and paired PBMC samples from three GBM subjects. After QC and normalization, M-MDSC samples were integrated, and data from 12,411 cells was clustered. To align with two-compartment deconvolution (Neu-like, DC-like), 2-cluster models were created, which was also supported by a high average adjusted Rand index (ARI >.85). Cluster 0 expression was defined with genes such *as NAMPT, SAMSN1, S100A12* and *S100A8*, and cluster 1 was defined by *MTSS1, ID2, HLA-DRA, and HLA-DPA1* (Fig. 6a). We define cluster 0 and cluster 1 in this 2-state model as Neu-like *(S100A8/A9*) and DC-like (*HLA-DR, CD74, ID1/ID2*), respectively. These classifications were done based on the marker genes from each cluster, as well as creating NeuMo and DC-like gene expression module scores, which showed the mapping of cluster 0 to a Neu-like state and cluster 1 to a DC-like state (Fig. 6b). At the 2-cluster level, Neu-like and DC-like clusters were observed in approximately 70%/30% proportions. Using paired PBMC samples, data were integrated and clustered, and cell type prediction performed using Azimuth (Fig. 6c). We extracted only the CD14^+^/CD16^−^ monocytes and predicted the cell type identity using the M-MDSC 2-cluster models as the references. Across a four-experiment average, the Neu-like transcriptional states (i.e., predicted cluster 0) were a lower fraction (38%) compared to the M-MDSC (68%) (Fig. 6d). The cells predicted to be cluster 0 were also those cells with the highest NeuMo module score (Fig. 6e). In each of the three paired samples, M-MDSC Neu-like fractions were greater than CD14^+^ monocytes (Fig. 6f) (p<0.001).

**Figure 7.**
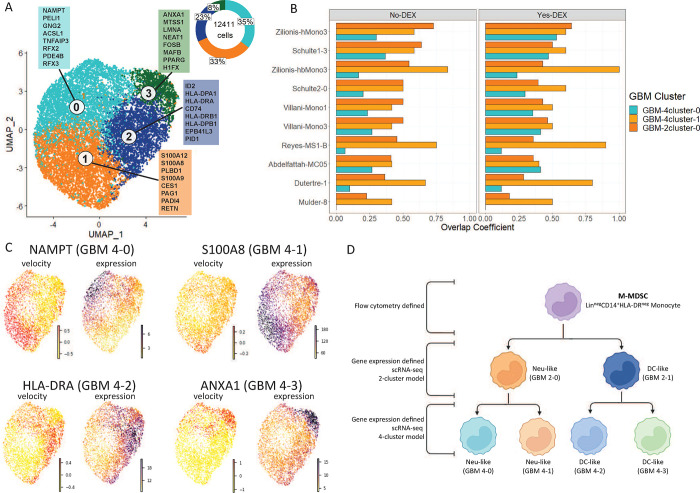
Integrating GSEA analyses including 80 published gene sets with 4-cluster resolution GBM scRNA-seq as applied to differentially expressed genes in bulk sequenced M-MDSC. **A.** Sub-clustering of the M-MDSC 2-cluster model. Cluster 0 from Fig 6A is split into cluster 0 and 1. Cluster 1 from Fig. 6A is divided in cluster 2 and 3. The boxes represent the top 8 marker genes by log_2_FC for each of the four clusters. **B.** Bar plots showing the overlap coefficient between Neu-like GBM single-cell clusters and various Neu-like literature-derived single-cell clusters. The overlap coefficient between two clusters is computed by comparing the leading-edge genes for each cluster from the GSEA analysis with bulk RNA-seq data. GBM-2cluster-0 is cluster 0 from Fig6A. GBM-4cluster-0 and GBM-4cluster-1 are cluster 0 and 1 from Fig 7A. **C.** UMAPs showing the RNA velocity and expression of a representative marker gene for each of the 4 clusters in A for one of the M-MDSC samples. **D.** Schema of M-MDSC heterogeneity.

Based on ARI (>0.83), indicating the stability of 4-cluster models, we split the 2-cluster model into a 4-cluster model revealing further heterogeneity and two Neu-like (GBM 4 cluster 0, GBM 4 cluster 1) and two DC-like transcriptional states (Fig. 7a).

### The similarity of published single--cell states with scRNA-seq GBM M-MDSC.

Using the cluster marker genes of each GBM cluster (Supplementary Table 10), another GSEA with the bulk RNA-seq M-MDSC/monocyte data was performed (Supplementary Tables 11, 12). The GBM scRNA-seq marker clusters were integrated with published studies by computing the overlap coefficients for each pairwise comparison of the total 86 clusters (Supplementary Tables 13, 14, Supplementary Fig. 4). The overlap coefficient was calculated using the sets of leading-edge genes from the GSEA. The NeuMo cluster marked by *S100A8/9/12* (GBM-4cluster-1) showed a greater overlap coefficient with four published monocyte clusters (Zilionis hbMono3, Reyes M1-B, Duterte-1, Mulder 8) compared to the 2-cluster model suggesting refined subcluster definition (Fig. 7b). The similarity of “GBM-4cluster-0” with published data was reduced compared to literature data suggesting GBM unique transcripts. RNA velocity estimates confirmed the similarity of mRNA processing in marker genes used to define GBM subclusters (Fig. 7c, Supplementary Fig. 5). Based on these results and published work, we propose a scheme to understand M-MDSC heterogeneity based on the putative dual lineage of human monocytes (Fig. 7d).

## Discussion

Using the results of scRNA-seq studies in healthy and diseased subjects, we interrogated bulk RNA-seq data from isolated M-MDSCs. We found a consensus transcriptional phenotype that embodies a neutrophil-like monocyte, or Neu-like, state. By using an independently derived marker gene set to deconvolute neutrophil-like monocytes, we reinforced our conclusion that M-MDSC gated cells in GBM are enriched in this transcriptional program. Finally, single-cell analyses confirmed higher Neu-like transcriptional clusters in isolated M-MDSC in GBM subjects. Further attesting to the robust nature of these associations, we confirmed greater Neu-like expression in M-MDSC of lung and head and neck cancer subjects. While much remains to be learned about the Neu-like monocytes, we note that M-MDSC displayed canonical gene expression and epigenetic marks (DNA methylation) of normal monocytes and not those of granulocytes. This argues against artifactual contamination of our M-MDSC cell isolates with neutrophils. Instead, these results indicate that a large portion of M-MDSCs are an outgrowth of an alternate Neu-like monocyte ontogenic pathway. The Neu-like and DC-like monocyte states in mice have been traced to different bone marrow progenitors (i.e., GMP and MDP, respectively)^46,47^. However, GMP fate-mapping and other evidence led to an alternative model wherein GMPs give rise to cMoPs and Neu-like monocytes, whereas MDP supports DC-like pools of cells^48^. Thus, while the existence of Neu-like and DC-like monocytes in mice is well established, the exact developmental intermediates and branching points between GMP and MDP progenitors remain to be clarified. The analogous human developmental schemes are less well studied^47^.

The predominant enriched transcriptional clusters in human GBM M-MDSCs corresponded to previously observed mononuclear phagocyte states marked by *S100A8*/*9*/*12*^49,51–55^, which were clinically significant in severe COVID-19, bacterial sepsis, lung cancer, and GBM tissue. In contrast, negatively enriched states exhibited MHC class II, complement, and related antigen presentation transcripts and were designated DC-like. However, there was an exception to this alignment in one previously reported DC-like cluster. Our similarity matrix (Supplementary Fig. 4) showed a close relationship between our GBM Neu-like clusters with Villani et al.^49^ DC3 and Dutertre et al.^60^ cluster cMo1. Dutertre et al.^60^ previously noted the similarity of DC3 to their classical monocyte cMo1. They proposed that the comparison of DC3 with other DC-like cells and not with monocytes, as was done by Villani et al.^49^, led those researchers to designate DC3 as a dendritic cell. Consistent with this latter interpretation, in our data, Dutertre cMo1 and Villani DC3 were significantly enriched in M-MDSC and contained many overlapping Neu-like genes. Thus, we saw a consistent core of transcripts demarcating putative neutrophil and dendritic-like monocytes.

When juxtaposing our findings with the current classification of healthy monocytes as classical, intermediate, and non-classical based on CD14 and CD16 cell surface expression^61^, we observed a dramatic association of Neu-like transcription with classical versus non-classical monocytes. Depending upon the experiment, classical monocytes (CD14^+^CD16^low^) displayed approximately 25–40% Neu-like and 60–75% DC-like transcriptional features, whereas non-classical monocytes (CD14^low^CD16^+^) were predominately DC-like (i.e., 90–96%). Given that non-classical cells are thought to be derived from classical monocytes, these results suggest they arise from a distinct transcriptional subtype of DC-like cells. In M-MDSCs, the DC-like cluster was detected at lower abundance (i.e., approx. 30%) compared with paired HLA-DR^+^ monocytes. While still detectable in MDSC, MHC class II transcripts were expressed at significantly lower levels compared to paired HLA-DR^+^ monocytes, which is expected, as M-MDSCs are sorted based on their negative surface HLA-DR staining. The functional properties of DC-like M-MDSCs are uncertain. However, subcluster 3 (Fig. 7a) of these cells was marked by *ANXA1*, a gene implicated as a mediator of tumor immunosuppression^39^ and a previously proposed marker of M-MDSC.

We and others have found that DEX treatments in glioma are associated with elevated M-MDSC concentrations in blood^14,20^. From the current study, we can add that the proportions of Neu-like clusters were increased in glucocorticoid-exposed MDSCs and paired monocytes from GBM subjects. Neu-like states represented up to 78% of M-MDSCs from GBM subjects exposed to DEX compared to only 13–40% in healthy, non-glucocorticoid-exposed donor total monocytes. The influence of glucocorticoids was confirmed by the greater Neu-like fractions in monocytes from subjects with autoimmune giant cell arteritis treated with prednisone compared to untreated patients or healthy controls^57^. The nature of DEX influence on differentially expressed genes that discriminated M-MDSCs from paired monocytes appeared quantitative rather than qualitative. The drug affected fold-change measurements of expression levels of M-MDSC-related genes but did not alter their identity. This was evident in the significant overlap of gene enrichment and functional pathways in DEX-exposed and non-exposed subjects. We use the term attenuation to describe the effect of DEX on differential gene expression. *CD39/ENTPD1*, which encodes ectonucleoside triphosphate diphosphohydrolase 1, the rate-limiting ectoenzyme that controls microenvironmental ATP concentration and is critical to initiate and maintain immune cell activation^66^, serves as an example. In non-exposed cells, *ENTPD1* transcripts were significantly higher in M-MDSCs compared to paired non-exposed monocytes. However, transcript levels were increased in paired monocytes in DEX-exposed subjects. The net effect of DEX was to reduce the differential expression of the gene and loss of statistical significance. A similar phenomenon was found for many other genes, as the drug influenced both cell populations differently. Thus, glucocorticoids as a drug class modify gene expression directly and bias the transcriptome of monocytes and M-MDSC towards a Neu-like state by altering the proportions of transcriptional states within monocyte populations. The possibility that DEX mediates the expansion of an alternate pathway of monopoiesis adds yet another dimension to the complex effects of glucocorticoids on the immune system.

Myeloid cells constitute the dominant immune component of the GBM tumor microenvironment (TME)^55,63,64^. It is still a source of speculation as to which of the heterogeneous transcriptional states of circulating myeloid cells now identified contribute to tumor-infiltrating populations. Here, we noted the enrichment of NeuMo gene transcripts in GBM tissues^55^. Arguing in favor of Neu-like expression as a bridge between blood and the TME were previous results in lung cancer^54^ that found high concordance of the hbMono3 transcriptional state in blood with hMono3 in lung tumors^54^. In GBM, we discovered that hbMono3 was enriched in bulk-sequenced M-MDSC and contained many overlapping genes with scRNA-seq GBM NeuMo clusters. Genes marking the “GBM-4 cluster-1” (Fig. 7b) completely overlapped and were a proper subset of the hbMono3 gene set. An earlier scRNA-seq study^55^ of bone marrow--derived myeloid cells in glioma reported five specific myeloid gene signatures (MC2–MC5 and MC7) as independent prognostic indicators of glioma patient survival. The MC5 state was described as a pro-tumorigenic macrophage with high expression of the alarmins, including *S100A4*. In non-DEX exposed subjects’ M-MDSC, we observed enrichment of the MC4 and MC5 clusters, although *S100A4* was not observed. We also found NeuMo scores to be strongly associated with low tumor purity. Earlier studies showed low tumor purity reflected bone marrow-derived myeloid infiltration related to poor patient survival^65^. This suggests that NeuMo expression signals the myeloid contributions within the TME. In TCGA and CGGA analyses, tumor purity and NeuMo scores were associated with survival in univariate analyses. NeuMo scores achieved statistical significance in multivariate survival models of IDH WT glioma, indicating the clinical relevance of NeuMo transcriptional signatures. In TCGA data when both NeuMo scores and tumor purity were included neither remained statistically significant.

To elucidate bulk-sequenced transcripts supporting M-MDSC effector functions of therapeutic import, we queried known immunoregulatory gene transcripts in M-MDSC^31^. Not unexpectedly, negative fold-change estimates were observed for transcripts encoding costimulatory proteins. Consistent with other studies of human M-MDSC^42^, we did not find increased expression of arginase (*ARG1*) or many immunomodulatory therapeutic targets (e.g., immune checkpoints). The aforementioned *ENTPD1* transcripts were overexpressed in No-DEX but not in Yes-DEX subjects. *ENTPD1* encodes ectonucleoside triphosphate diphosphohydrolase 1, the rate-limiting ectoenzyme that controls microenvironmental ATP concentration and is critical to initiate and maintain immune cell activation^66^. After considering false discovery to focus on the most generalizable immunomodulatory targets, we prioritized genes over-expressed in both DEX naïve and DEX exposed cells. Two genes meeting these criteria were *Regnase-1* and *TNFAIP3. TNFAIP3* was associated with M-MDSC in HNSCC but not in NSCLC. Regnase-1 was overexpressed in both NSCLC and HNSCC as well as being associated with glioma molecular subtype, tumor purity and survival.

Because *Regnase-1* transcripts were not detectable in GBM scRNA-seq clusters, we could not map them to a specific subcluster. However, they were highly correlated with NeuMo gene expression (R=0.78) in bulk sequencing and, like NeuMo scores, were associated with glioma molecular subtype and survival in TCGA and CGGA data. The differential expression of *Regnase-1* in M-MDSC was not attenuated by DEX treatment even though it contains glucocorticoid receptor binding sites and cooperates with the drug in regulating inflammation^56^. The gene product of *Regnase-I* is an RNA-binding endoribonuclease and deubiquitinase that plays a critical role in inflammation by targeting mRNA stem-loop structures and degrading transcripts of inflammatory cytokines (e.g., IL-6, IL-1β, ICOS)^67,68^. By controlling RNA stability, Regnase-1 joins a growing family of RNA binding proteins^69,70^, promising drug-able targets in immunity. Multiple strategies have evolved to modify RNA binding proteins to enhance anti-cancer immunotherapies^71^; most have focused on T and B adoptive cell therapies^72–74^. Targeting myeloid populations in the CNS has received less attention. However, the antisense-mediated loss of Regnase-1 function in brain microglial cells prevented neuroinflammation and neuronal damage^75^. Intracranial delivery of antisense oligonucleotides targeting stem-loops in Regnase-1 mRNA achieved clinical benefit in mouse experimental autoimmune encephalitis (EAE). Modulating Regnase-1 in EAE suppressed proinflammatory cytokines, prevented bone marrow-derived myeloid cell recruitment, and modified resident microglia^76^. The mucosal-associated lymphoid tissue gene (*MALT1*), a negative regulator of Regnase-1 and other RNA binding proteins, was shown to regulate glioma cell survival^77^. Another potential drug-able target, identified as a NeuMo “GBM-4cluster-0” marker, was nicotinamide phosphoribosyl transferase (*NAMPT*)^78^. NAMPT is an active area of small molecule drug development^79^. The *NAMPT* gene product has been implicated in mobilizing MDSCs^78^ and targeting the degradation of NAMPT-augmented antitumor immunity in an animal model^80^. Transcripts of other immune-modulating genes (*PELI1*^81^, *ANXA1*^82^, *MAFB*^83^) were identified as GBM subcluster markers.

The resemblance of the neu-like monocyte state to MDSCs was alluded to recently^47,84^, but the implications of these observations have not been explored in human glioma. Our results indicate that the concept of a dual ontogeny of human monocytes and M-MDSC may be helpful in GBM and provide a conceptual framework for understanding the heterogeneity of these cells that has eluded investigators. This may have implications in other malignancies, including lung and head and neck cancer. Even broader applications are suggested by the similarity of M-MDSC transcriptional states with those observed in COVID-19 and bacterial sepsis. Our results help elucidate the heterogeneity of the M-MDSC transcriptome and support a novel hypothesis that M-MDSCs are at least partly derived from a newly described monocyte development pathway associated with cancer, severe infection, and glucocorticoid exposure.

## METHODS

### Patient and control samples.

Monocytes and M-MDSC were isolated from the UCSF Immune Profiles Study (IPS) volunteers, a prospective neurosurgery and neuro-oncology clinic-based collection of blood samples, imaging, and other clinical data from adult glioma patients. All studies were approved by the Institutional Review Board of the University of California, San Francisco, Human Research Protection Program in the Office of Ethics and Compliance under UCSF Federal-wide Assurance 00000068 and met all relevant ethical regulations. Informed consent was obtained from all study participants. Presurgery blood samples were typically taken the day before surgery; none were obtained during or after exposure to anesthesia. Blood samples were transferred the same day as drawn for fluorescence activated cell sorting (FACS) solation and bulk and scRNA studies. We collected a questionnaire during blood draws to document daily/cumulative DEX exposure. We designated GBM according to the WHO 2021 classification as IDH wildtype grade 4 astrocytoma.

### FACS isolation of M-MDSCs and HLA-DR^+^ monocytes.

Fresh anticoagulated blood was processed within 24 hours. Blood mononuclear cells were isolated with 1.077 Histopaque gradients, stained with a cocktail of fluorescently labeled antibodies (CD3, CD56, CD19, CD14, CD11b, CD16, HLA-DR, CD33, CD66b and CD15^20^ (Supplementary Fig. 6, Supplementary Table 18), treated with PE/Cyanine7 Streptavidin and resuspended at 1:5000 dilution of SYTOXTM Green. Cells were then run directly on a BD FACSAriaTM Fusion cell sorter. Forward scatter hi CD3− CD56− CD66b− Side scatter low CD11b+ CD33+ CD14+ CD15− monocytes were gated and plotted for HLA-DR expression. CD3 HLA-DR^−^ neg cells and CD19 HLA-DR^+^ positive B cells were used to set the sorting gate for M-MDSC cells lacking HLA-DR expression (i.e., HLA-DR^neg/low^). HLA-DR^high^ cells (normal monocytes) were collected from the same individuals. In some subjects, the HLA--positive CD14 monocytes were collected as two fractions representing the uppermost 20% in HLA-DR expression versus the bottom 80% yielding HLA^pos^ and HLA-DR^hi^ fractions. The purity of isolates was checked using CIBERsort expression and a high-definition immune cell methylation deconvolution method^58^. To compare with conventional monocyte designations, classical, intermediate, and non-classical monocyte subtypes were isolated from 8 healthy subjects (1:1 male: female) using a combination of MACS (magnetic-activated cell sorting) and FACS. Briefly, leukoreduction system chambers were obtained and from the local blood donation center, back-flushed, and PBMCs were collected by Ficoll-Paque PLUS (Cytiva 17–440-02) gradient. Samples were enriched for monocytes using pan-monocyte MACS negative selection (Miltenyi kit #130–096-537) to deplete the bulk of unwanted cells. The resulting pan-monocyte enriched cells were fluorescently labeled and cell sorted into monocyte sub-populations: classical (CD14++, CD16−, HLA-DR^low^), intermediate (CD14+, CD16++, CD36+, CCR2+) and two non-classical subsets (SLAN+: CD14++, CD16+, HLA-DR+, SLAN+ CD14++, CD16+, HLA-DR+, SLAN−, CD36^low/−^, CCR2^low^) [See Supplementary Table 18 for antibody details]. The purity of isolated cells was 98% for classical, 71% for intermediate, and 95% for both SLAN − and SLAN + non-classical monocytes. All isolated cell pellets were stored at −80°C until DNA methylation or bulk RNA seq analyses.

### DNA and RNA isolation.

Total RNA and genomic DNA were isolated from 200–500 × 10^5^ monocytes or M-MDSCs using the AllPrep DNA/RNA mini kit according to the manufacturer’s instructions (Qiagen). RNA quality was assessed by bioanalysis (Agilent), with all samples having RNA integrity numbers > 9. Total RNA and genomic DNA concentrations were determined by Qubit^®^ 2.0 Fluorometer (Life Technologies, Carlsbad, CA, USA).

### DNA methylation deconvolution.

DNAm preprocessing and cell deconvolution was performed as described^58^. Data from M-MDSC and monocytes from glioma subjects were combined with monocyte (N=5) and neutrophil (N=6) data from healthy subjects downloaded from the Flow.Sorted.Blood.EPIC Bioconductor package in R. The combined data were subset to CpG sites that define neutrophil and monocytes in cell mixtures. A heatmap was used to visualize these cell types’ methylation status at monocytes and neutrophils’ canonical epigenetic features.

### RNA extraction and stranded RNA-seq library preparation.

RNA samples (200 ng total RNA) that passed quality checks were used as input to KAPA RNA Hyperprep with RiboErase (Roche) library kits. Briefly, ribosomal RNA was depleted with RNase H and mRNA was enriched via polyA selection from input total RNA. Enriched mRNA was then fragmented, followed by first-strand cDNA synthesis with random priming and second-strand cDNA synthesis with dUTP. The 3′ adenylates were added to the double-stranded cDNA, followed by adaptor ligation and second--strand removal and amplification. Libraries were sequenced using the Illumina HiSeq2500 instrument (Illumina) to generate paired--end reads (2 × 100). The sequencing depth was approximately 40 million reads per sample, and an average of 14,000 detected genes.

### RNA sequencing data pre-processing.

Sequence read quality was assessed with FastQC (v0.11.8; http://www.bioinformatics.babraham.ac.uk/projects/fastqc/). Reads were mapped, and transcript abundance was quantified at the gene level using RSEM (v1.3.1) with the bowtie2 aligner (v2.3.5.1) and the UCSC hg38 human reference assembly.

### Differential expression analysis.

Differential gene expression analysis used the Bioconductor package edgeR (v3.36.0). Genes with low expression across all libraries were removed from the analysis, keeping only genes that expressed more than one count per million (CPM) in more than 3 samples. Paired M-MDSC and monocyte samples from patients taking DEX at blood draw (Yes-DEX, N=6) and not taking DEX at blood draw (No-DEX, N=12) were tested separately. The quasi-likelihood negative binomial generalized log-linear model was used to test for differential expression between M-MDSC and monocyte samples, considering their paired nature. The magnitude of the difference was calculated as the log_2_ transformed fold-changes of M-MDSC vs. monocyte. Differentially expressed genes were determined using a Benjamini-Hochberg false discovery rate (FDR) < 0.05.

### DEX Attenuation.

To identify common differentially expressed genes (DEGs) between M-MDSCs and monocytes in the presence or absence of DEX, we compared the log_2_ fold-changes (FC) of DEGs in both groups. We found 666 genes differentially expressed in the same direction in both groups. The ratios of the log_2_-FC for each common gene pair between the Yes/No DEX groups were calculated. If the ratio was less than 1, the gene was considered to have undergone DEX attenuation. Conversely, if the ratio was greater than 1, the gene was deemed to have undergone DEX potentiation. To assess whether the number of genes with DEX attenuation was significant, a null distribution was created by randomly sampling 666 genes from a set of ~13000 genes with log_2_-FC in the same direction, computing their Yes-DEX to No-DEX ratio of log_2_-FC and counting the number of ratios less than 1. This process was repeated 100,000 times, and the resulting distribution of the ratios less than 1 was compared to the observed number of ratios less than 1.

### Pathway Analysis of DEGs and DEX attenuated genes.

Pathway analysis was performed for the Yes-DEX DEGs, No-DEX DEGs, and DEX attenuated genes. The Overrepresentation analysis (ORA) method was used with QIAGEN Ingenuity Pathway Analysis (IPA) and the Gene Ontology (GO) biological processes with the enrichGO function in the clusterProfiler R package. To simplify the output by removing redundant enriched GO terms, the simplify function in the clusterProfiler R package was used. An IPA Canonical Pathway or GO biological process was considered significantly over-represented if the p-value< 0.05.

### Identifying scRNA-seq studies.

A literature review was conducted to identify single-cell RNA-sequencing (scRNA-seq) studies in which myeloid cell populations in inflammatory conditions/diseases were defined. Studies were included if the list of cluster-specific marker genes for each myeloid cell population was easily accessible and interpretable (Supplementary Table 4).

### Gene set enrichment analysis.

A gene set enrichment analysis was conducted using log_2_-FC values from the differential expression analysis and the myeloid cell population marker genes from scRNA-seq studies with the WebGestalt online tool (http://www.webgestalt.org/). Each cell-specific cluster’s list of marker genes was treated as its own gene set (uploaded under “Function Database” on WebGestalt), and the log_2_FC values for every gene tested for differential expression were input as the gene list (uploaded under “Gene List” on WebGestalt). Yes-DEX and No-DEX genes were tested separately. The output is an enrichment score indicating whether each gene set (i.e., cell-specific marker genes) is enriched with up-regulated or down-regulated genes in M-MDSCs compared to monocytes.

### NeuMo gene expression score.

For the No-DEX and Yes-DEX groups, gene sets with enrichment of up-regulated genes in M-MDSCs were identified (normalized enrichment score ≥ 2.5 and FDR ≤ 0.05). These gene sets’ leading-edge genes were compared to find genes in most gene sets (a gene was in ≥50% of the enriched gene sets). Thirty-nine genes were found at the intersection between the No-DEX and Yes-DEX groups (N=39 genes). This intersection of genes is the basis of the NeuMo score. The NeuMo score is the average log_2_ counts per million (CPM) of those 39 genes.

### Pathway analysis of NeuMo genes.

To identify canonical signaling pathways and biological processes from the genes that make up the NeuMo score, the set of NeuMo genes was expanded. The enlarged set included genes whose expression correlated positively with the NeuMo score at a Pearson correlation coefficient of 0.7 or higher. Overrepresentation analysis (ORA) was performed using the Gene Ontology (GO) biological processes with the enrichGO function in the clusterProfiler R package. To simplify the output by removing redundant enriched GO terms, the simplify function in the clusterProfiler R package was used. A GO biological process was considered significantly over-represented if the p-value< 0.05.

### External datasets for assessing NeuMo score.

Three publicly available datasets from the Gene Expression Omnibus (Supplementary Table 15) containing bulk RNA-seq were used to assess the NeuMo scores for isolated M-MDSCs and monocytes. An HNSCC (GSE183854) dataset that has RNA-seq : for five isolated M-MDSCs from HNSCC patients and five isolated monocytes from HNSCC patients. An NSCLC (GSE162353) dataset that has RNA-seq for 3 isolated monocytes, and 3 isolated M-MDSC samples from NSCLC patients. A giant cell arteritis dataset (GSE201753) that has RNA-seq for 29 isolated monocytes from healthy individuals, 33 isolated monocytes from individuals in remission treated with prednisone, and 29 isolated monocytes from individuals in remission not treated with prednisone. A dataset with bulk RNA-seq in whole blood from patients with glioblastoma (GBM) from Qi et al. was also utilized^85^. This dataset includes RNA-seq in whole blood from 10 GBM patients and 12 non-GBM donors. To assess the NeuMo score in a dataset with bulk RNA-seq in tumor tissue from patients with glioma, the publicly available data from The Cancer Genome Atlas (TCGA) and the Chinese Glioma Genome Atlas (CGGA) were used. For TCGA, the counts files for the GBM and LGG projects were downloaded using the GDC data portal. The log_2_(CPM) values were calculated from count data. This dataset includes RNA-seq for 702 tumor samples, of which 684 have IDH mutation status and survival data available and were used for downstream analysis. For the CGGA, two datasets were downloaded: the read counts from mRNAseq_693 (batch 1) and mRNAseq_325 (batch 2). The two-count matrices were combined, and the log_2_(CPM) values were calculated. Batch correction was conducted using the ComBat function in the sva Bioconductor package. The covariate for tumor grade (2, 3, or 4) was included in the batch correction. This dataset contains RNA-seq for 1013 tumor samples, of which 885 have IDH mutation status available and are used for downstream analysis.

### Semi-supervised NMF deconvolution of Neu-like and DC-like monocytes.

A semi-supervised non-negative matrix factorization (NMF) deconvolution algorithm called NITUMID was used to deconvolute M-MDSC and monocyte RNA-seq samples from GBM (this study), HNSCC (GSE183854), NSCLC (GSE162353), GCA and healthy (GSE201753) donors. Here, the semi-supervised NMF algorithm makes use of 14 marker genes to guide the factorization/deconvolution process and deconvolute a sample into Neu-like and DC-like fractions. The guide matrix for input into the NITUMID method was created by coding genes based on their expression level in a cell. A value of “1” indicates the gene is highly expressed in a cell; and a value of “0” indicates the gene is not expressed in the cell. Marker genes were selected from two cell types identified in Weinreb et al.^47^ (Neu-like and DC-like monocytes) using a log_2_ (fold-enrichment) cutoff of 0.58. Genes that pass this cutoff for the Neu-like monocytes are given 1 and 0 for the DC-like monocyte cell type. And vice-versa for genes that pass the cutoff for DC-like monocytes (Supplementary Table 16). The NITUMID algorithm was run using the R package on GitHub (https://github.com/tdw1221/NITUMID).

### Assessment of NeuMo score in isolated monocytes and M-MDSCs.

The NeuMo score was calculated for our M-MDSCs and monocytes isolated from glioma patients, as well as for all the samples in the HNSCC (GSE183854), NSCLC (GSE162353), and GCA (GSE201753) datasets. To obtain a pooled estimate of the mean difference in NeuMo score between M-MDSCs and monocytes, a meta-analysis with a fixed-effect model was performed with the glioma, HNSCC, and NSCLC data using the metacont function in the meta R package. Differences in NeuMo score between Yes-DEX and No-DEX monocytes and M-MDSCs were measured using Wilcoxon rank sum tests. Differences in NeuMo score between prednisone--exposed monocytes from healthy and GCA donors were measured using Wilcoxon rank sum tests. A p-value<0.05 was considered statistically significant.

### Assessment of NeuMo score in whole blood.

NeuMo score was calculated for whole blood samples from a GBM and non-GBM donor study. First, CIBERSORTx with the LM22 signature matrix was run in absolute mode to deconvolute the whole blood samples. Then, a linear regression model was fitted, modeling the NeuMo score as the dependent variable and condition (GBM or non-GBM) and neutrophil level (obtained by CIBERSORTx) as the independent variables.

### Assessment of Neu-like deconvolution fraction in isolated monocytes and M-MDSCs.

From the semi-supervised NMF deconvolution, the Neu-like fraction was compared across the GBM, HNSCC, NSCLC, and GCA datasets in the same way as the NeuMo score was assessed.

### Survival analysis in tumor tissue.

Using all glioma samples, the NeuMo score was dichotomized into a high NeuMo score group and a low NeuMo score group. The TCGA samples (N=684) served as the training set, and the R package partDSA was used to determine the cutpoint at which the NeuMo score was partitioned. Individuals with a NeuMo score above the cutpoint fall into the high NeuMo score partition, and those below are in the low NeuMo score partition. The same cutpoint was applied to the CGGA samples (N=885), serving as a validation set. Kaplan-Meier survival curves and log-rank tests were used to visualize and determine the association between the NeuMo score group and survival. To conduct a multivariate analysis, Cox proportional-hazards (PH) models were fit independently to the TCGA and CGGA for the NeuMo score group and adjusted for IDH mutation status, tumor purity (measured with consensus purity estimation (CPE) method is only available for the TCGA), age, and grade. Models were fit in R using the coxph function. The PH assumption was tested using Schoenfeld residuals. Since IDH status violated the PH assumption, it was fit as a stratum in the model. Both the TCGA and CGGA data were subset to only IDH WT tumors, and in the same way, the NeuMo score was dichotomized into groups using the TCGA as the training set and the CGGA as the validation set. Cox PH models were fit to the TCGA IDH WT and CGGA ID WT data with the NeuMo score group as a predictor and adjusted for tumor purity (only TCGA), age, and grade. A p-value < 0.05 was statistically significant.

### Single-cell RNA sequencing from GBM subjects’ PBMCs and M-MDSCs.

Cell sorting and library creation were performed by the UCSF Flow Cytometry and Genomics CoLabs, respectively (San Francisco, CA). PBMCs and FACS-sorted M-MDSC populations were normalized to 1000 cells/ul suspensions in 0.04%BSA/1x PBS. Twenty-five thousand cells were loaded onto the 10X Chromium System (10X genomics) and encapsulated using the Standard Chip. Single-cell Dual index 3’v3.1 Gene Expression Libraries were generated according to the manufacturer’s instructions. Completed libraries were sequenced on the NovaSeq 6000 S4 (Illumina) platform at a targeted median read depth of 20,000 paired reads per cell. Raw sequencing reads were aligned to GRCh38 (human) using Cell Ranger (v.7.1.0) software with default parameters. Subsequently, genes were quantified as UMI counts using Cell Ranger and initially visualized using the Cell Ranger web summary. Downstream analysis was performed on filtered feature counts generated by Cell Ranger. Low-quality single cells containing <2000 or >5000 expressed genes or <0.8 log_10_(Genes/UMI) or >5% mitochondrial transcripts were removed. Additionally, genes expressed in fewer than 10 single cells were removed. We identified and removed potential single-cell doublets using scDblFinder (v1.8.0) with the default settings. Using Seurat (v4.1.3), each sample was normalized using the “LogNormalize” method, and the 2000 top variable features were chosen using the “vst” method. Then, M-MDSC and PBMC samples were integrated using Seurat’s (v4.1.3) integration methods. For the M-MDSC samples, only cells predicted to be CD14+ monocytes by the Azimuth program were kept for final clustering. The final clustering solution for M-MDSC samples was determined by finding the optimal number of principal components (nPCs) and resolution were determined by assessing the robustness/stability of clusters. Briefly, for the nPCs chosen by the elbow method and for a specific/given resolution, 1) run the initial clustering solution at a set random seed, 2) run clustering 100 more times at that resolution, each time, with a different random seed, 3) compare clustering solution labels between original clustering from step 1 to all subsequent iterations by computing the adjusted Rand index (ARI) 4) repeat steps 1–3 by increasing the resolution by 0.05. Clustering was performed with the Louvain algorithm using resolutions from 0.1 to 0.5, and the optimal resolution was chosen to be the one where the ARI began to decrease. For M-MDSCs, the final clustering was defined with a resolution of 0.15, resulting in 2 clusters.

### Sub-clustering of M-MDSC.

The ARI also indicated the stability of 4 clusters, so the FindSubCluster function in Seurat was used to split the 2-cluster M-MDSC model into smaller clusters. Each original cluster was divided into two smaller clusters with this function, resulting in a 4-cluster M-MDSC model.

### Differential expression analysis of M-MDSC clusters.

Differentially expressed genes (i.e., markers of clusters) were determined for each cell cluster by a Wilcoxon rank-sum test that compares cells in a cluster to all other cells. Marker genes were defined to be expressed in at least 25% of cells, have a log_2_FC > 0.25, and an adjusted p-value<0.05. For visualization, UMAP projections were computed on that dataset’s optimal number of PCs. This was done independently for the 2-cluster and 4-cluster M-MDSC models.

### Gene expression module scores.

The AddModuleScore function in Seurat was used to compute a NeuMo and DC-like gene expression module score. For the NeuMo module, the 39 NeuMo genes were used as features for the expression program. For the DC-like module, the 8 DC-like genes used in NMF deconvolution were used as features for the expression program.

### Transfer Labels to predict clusters in PBMC CD14 Monocytes.

The PBMC data were clustered using the Seurat default settings. The data were subset to only CD14+ monocytes indicated by Azimuth. This CD14+ monocyte data was re-clustered using the method described above for M-MDSCs. Seurat’s transfer label’s method was used to determine the cell type identity of the CD14+ monocyte cells, according to our M-MDSC 2-cluster model. The reference group was the 2-cluster model built in the M-MDSC data, and the query group was the CD14+ monocytes from PBMC.

### Overlap coefficient of single-cell gene sets.

The GBM M-MDSC 4-cluster and 2-cluster gene sets were subjected to a GSEA as described earlier when analyzing the 80 published single-cell gene sets. The leading-edge genes from the GSEA from all 86 gene sets were used to compute the overlap coefficient for all pairwise comparisons.

### RNA Velocity.

The velocyto and scVelo pipelines were used for RNA velocity analysis. Analysis was done in Python (v.3.9.6).

## Data Availability

Methylation and phenotype data used in this manuscript are available through dbGaP--controlled access. Methylation and phenotype data from the Immune Profiles Study are available through dbGaP Study Accession phs002998.v1.p1 (https://www.ncbi.nlm.nih.gov/projects/gap/cgi-bin/study.cgi?study_id=phs002998.v1.p1). Source data files have been provided with this manuscript.
